# Navigating Climate Adaptation on Public Lands: How Views on Ecosystem Change and Scale Interact with Management Approaches

**DOI:** 10.1007/s00267-020-01336-y

**Published:** 2020-07-29

**Authors:** Katherine R. Clifford, Laurie Yung, William R. Travis, Renee Rondeau, Betsy Neely, Imtiaz Rangwala, Nina Burkardt, Carina Wyborn

**Affiliations:** 1 USGS-Fort Collins Science Center, Social and Economic Analysis Branch, Fort Collins, CO USA; 2 Western Water Assessment, University of Colorado, Boulder, CO USA; 3 North Central Climate Adaptation Science Center, Boulder, CO USA; 4 Department of Society and Conservation, University of Montana, Missoula, MT USA; 5 Department of Geography, University of Colorado Boulder, Boulder, CO USA; 6 Colorado State University, Fort Collins, CO USA; 7 Colorado Chapter, The Nature Conservancy, Boulder, CO USA; 8 Institute for Water Futures, Fenner School of Environment and Society, Australian National University, Canberra, ACT Australia

**Keywords:** Transformation, Land management, Environmental change, Regime shift, Resilience and active intervention

## Abstract

Managers are increasingly being asked to integrate climate change adaptation into public land management. The literature discusses a range of adaptation approaches, including managing for resistance, resilience, and transformation; but many strategies have not yet been widely tested. This study employed in-depth interviews and scenario-based focus groups in the Upper Gunnison Basin in Colorado to learn how public land managers envision future ecosystem change, and how they plan to utilize different management approaches in the context of climate adaptation. While many managers evoked the past in thinking about projected climate impacts and potential responses, most managers in this study acknowledged and even embraced (if reluctantly) that many ecosystems will experience regime shifts in the face of climate change. However, accepting that future ecosystems will be different from past ecosystems led managers in different directions regarding how to respond and the appropriate role of management intervention. Some felt management actions should assist and even guide ecosystems toward future conditions. Others were less confident in projections and argued against transformation. Finally, some suggested that resilience could provide a middle path, allowing managers to help ecosystems adapt to change without predicting future ecosystem states. Scalar challenges and institutional constraints also influenced how managers thought about adaptation. Lack of institutional capacity was believed to constrain adaptation at larger scales. Resistance, in particular, was considered impractical at almost any scale due to institutional constraints. Managers negotiated scalar challenges and institutional constraints by nesting different approaches both spatially and temporally.

## Introduction

Public lands present an important opportunity for climate adaptation, especially in the United States Intermountain West, where public lands constitute almost half of the land area. Public lands provide important ecological, social, cultural, and economic benefits, especially for nearby communities, and climate change impacts can increase social vulnerability for people who depend on public land resources (Knapp 2011; McNeeley et al. 2017). Public lands are subject to long-term planning and monitoring, require public involvement in decision-making, and serve multiple communities and constituencies. However, efforts to develop and deploy climate adaptation actions on public lands face unique challenges (Ellenwood et al. 2012; Burkardt and Orth 2018; Peters et al. 2018). Communities, scientists, and managers may disagree about what constitutes appropriate and effective adaptation. Land managers struggle to develop climate adaptation actions that meet community needs, match available science and professional training, and comply with relevant policy and law (Archie et al. 2012).

The Intermountain West is expected to change in important ways as a result of climate change. Climate models project that the region will become significantly drier (Overpeck and Udall 2010; Garfin et al. 2014) and experience more extreme events (Tebaldi et al. 2006), from increases in wildfire severity and extent (Westerling et al. 2006) to more intense precipitation events (Wuebbles et al. 2014). The Intermountain West is already experiencing many of these changes, including more intense and longer lasting droughts (Gonzalez et al. 2018), increased wildfire frequency and area burned (Westerling et al. 2006), earlier snowmelt and runoff (Clow 2010; Fritze et al. 2011), and decreased snowpack (Mote et al. 2018) due to more wintertime rain (Knowles et al. 2006). Finally, downscaling climate models and projecting future ecosystem conditions is challenging, especially in mountainous areas (Cozzetto et al. 2011; Rangwala and Miller 2012), and there are uncertainties regarding the specific ways in which climate change will manifest at the local level.

Until recently, public land management agencies were charged with planning for climate change (see e.g., Executive Order 13514 2009; EO 13653 2013; EO 13693 2015) and identified climate adaptation as a goal. Even though many of the federal policies on climate adaptation were rescinded in 2017, climate adaptation practices continue to advance in public land management agencies and a wide range of approaches have been described in the scientific literature. However, many adaptation strategies have not been widely implemented due to lack of resources, risk aversion, limited understanding of how to operationalize them, and low confidence in their efficacy (Archie et al. 2012; Archie 2014). In this paper, we describe a study that employed in-depth interviews and scenario-based focus groups to learn how managers envision future ecosystem change, and how they view different management approaches in the context of climate adaptation.

## Public Lands and Climate Change Adaptation

The Intergovernmental Panel on Climate Change (IPCC) defines climate adaptation as adjustments in natural or human systems in response to observed or projected climate impacts (IPCC, C. C. 2001). More recently, adaptation has been recognized as a social-ecological process that is enabled and constrained by a diversity of physical, biological, social, and institutional factors (Moser and Ekstrom 2010). Thus, climate adaptation on public lands can be best understood in the context of ecological change, management imperatives, and institutional structure and culture.

Most public land management focuses on current and desired future conditions, best management practices, the flow of resources, such as timber, water, and wildlife, and the recreation experiences that users have come to expect (Cortner and Moote 1999). Adapting to climate change may require altering conventional approaches and even some of the foundational assumptions and logics that underlie public land management (Joyce et al. 2009). For example, established “best practices” maybe no longer be appropriate or effective when applied to current or future landscapes (West et al. 2009). Current restoration efforts maybe destined to fail if changing climatic conditions cannot support historic ecosystems (see Tahoe National Forest in Littell et al. 2012). While management approaches to climate change often focus on ecological systems, they also engage with social systems because they interact with livelihoods connected to resources from public lands, recreational opportunities, and valued species and ecosystems. Thus, these decisions not only have ecological consequences; they are also highly political (Pelling 2010; Eriksen et al. 2015; O’Brien and Selboe 2015).

Given the uncertainty about future climate conditions and how they will impact local ecosystems, the scientific literature on adaptation increasingly emphasizes robust and flexible approaches (Walker et al. 2013; Kwakkel et al. 2016). Robust adaptation is designed to function over a wide range of future conditions and to adjust in response to change. Since public land managers, especially in the Intermountain West, must contend with varied landscapes that respond differently to climate, many recommend a “toolbox approach”, utilizing a wide range of approaches that include both conventional strategies, such as fuel treatments to reduce wildfire severity, and more novel strategies, such as assisted migration of species (Millar et al. 2007). There is no one-size-fits all approach to adaptation that will work across all ecological and social contexts, so managers face difficult decisions regarding what might work best given uncertain projections, potentially contested objectives, and a suite of tested and untested strategies.

While there are many ways to formulate or categorize adaptation goals, some frameworks emphasize resistance, resilience, and transformation (Millar et al. 2007). Different scholars variously define these three goals, and the lines between them often blur (Walker et al. 2004). Alternate frameworks categorize adaptation according to resistance, resilience, and response options (Millar et al. 2007); persistence, directed change, and autonomous change (Fisichelli et al. 2016); resilience, transition, and transformation (Pelling 2010); observe, resist, and facilitate change (Aplet and McKinley 2017). While not necessarily agreed upon, the concepts of resistance, resilience, and transformation provide a useful starting point for examining the approaches that managers emphasize.

The goal of resistance is to slow or stop climate change impacts or ecosystem changes, and to preserve historic ecosystem states (Parker et al. 2000). In many cases, resistance not only tries to slow changes, but takes on a restoration lens, aiming to return ecosystems to past states (Aplet and Cole 2010). Because resistance is akin to swimming upstream, against the current of climate change, resistance is generally assumed to require intensive, active intervention in ecosystem processes, and is therefore typically limited to areas that are highly valued, ecologically, economically, or culturally. However, at the same time, some managers are drawn towards resistance because of social norms and policy language that suggest landscapes are more “natural” when they remain in their historic ecological state (Cole and Yung 2010). These decisions are also shaped by policy (Stephenson and Millar 2012) and risk aversion (Hagerman and Satterfield 2014). Generally, resistance strategies become more labor-intensive and less effective over time (Millar et al. 2007).

Resilience is variously defined, but at its core resilience allows for more change as compared with resistance. A resilient system can change and adjust in response to stressors or disturbance but does not shift into a fundamentally different state (Zavaleta and Chapin 2010). In other words, a resilient system does not irreversibly and fundamentally change states in the face of external perturbations (Holling 1973; Gunderson 2000). Further, while resilient systems are dynamic, they are able to maintain key functions and ecosystem services in the face of change. A system can be resilient without management intervention, or managers can actively intervene to try to build resilience. Resilience plays an important role in the way the Forest Service conceptualizes agency responses to climate change (Timberlake and Schultz 2017). Further, while the ambiguity of resilience makes it difficult for managers to operationalize, it also provides flexibility that some managers value (Timberlake and Schultz 2017). Cross-scale interactions, or panarchy, can influence the resilience of a system, as disturbances or management actions at smaller scales can accumulate and influence larger scales (Chaffin et al. 2016; Gunderson et al. 2017).

In the face of a regime shift, managers can take a hands-off approach and simply observe changes, or they can intervene and assist with transformation (Cole and Yung 2010). According to Chaffin et al. (2016), transformation involves deliberate, human-driven actions that are intended to push a system across a threshold and to a new state, involving shifts in the key processes and structures that govern that system. We use the term transformation to refer specifically to management interventions to assist ecosystems in transforming to a new state (e.g., a regime shift from a forest to a grassland). Transformation maybe appropriate when significant changes to ecosystem processes, organization, or elements are inevitable (West et al. 2009) and when anticipated changes might degrade ecosystem functions and/or services over the long term. In these cases, managers may choose to actively intervene to retain some semblance of a functioning system and avoid catastrophic loses (Millar et al. 2007).

An example of a transformation strategy after a catastrophic wildfire in Pinyon-Juniper woodlands of southwest Colorado includes developing a climate-smart seed mix that allows the site to transform into a functioning grass–shrub ecosystem and reduces the establishment of non-native invasive species like cheatgrass (Rondeau et al. 2017a). Transformation represents a significant departure from conventional management, which typically seeks to preserve historic or current conditions (Landres et al. 1999). Further, the strategies employed for transformation are often novel and include assisted migration, deploying genetically modified organisms, and changing disturbance regimes (Millar et al. 2007). Panarchy is also important to understanding transformation, as cross-scale interactions affect capacity for transformation and large-scale transformation may depend on interventions at smaller scales (Chaffin et al. 2016).

Some agency staff maybe resistant to transformation and to novel strategies for climate adaptation. Kemp et al. (2015) found that, in the context of climate change, public land managers leaned heavily on existing management strategies that they had previously utilized for projects unrelated to climate change. These existing strategies were perceived to be more feasible and better supported by the agency and the public and were not seen as requiring accurate projections about future climate change impacts. Interestingly, other studies show that Forest Service staff at the national forest and regional-level envision climate change as a new challenge requiring new approaches in contrast to district-level staff who emphasize existing management strategies (Laatsch and Ma 2015). Even non-governmental organizations maybe largely unwilling to consider transformation and instead focus on resistance to preserve historic species composition (Poiani et al. 2011). Further, because different approaches have different implications for the livelihoods, recreational opportunities, and social values associated with public lands, the decisions that agencies make in response to climate change occur in a highly politicized context (Archie 2014).

In this study, we envision resistance, resilience, and transformation as management approaches that can sometimes overlap or complement one another. We see active intervention and hands-off management (i.e., no intervention) as opposites but emphasize that active intervention can be employed in many different ways and used to support different adaptation approaches (See Table 1). In the face of climate change, resistance is only possible through active intervention, but a hands-off approach could emphasize resilience and might be taken in the face of a regime shift.

**Table 1:** Types of management intervention

The solid line separates Active Management from approaches that often respond specifically and explicitly to climate change

The dashed lines recognize the blurry boundaries between resistance, resilience, and transformation

## Methods

### The Gunnison Basin

This research was conducted in the Upper Gunnison River Basin, referred to hereafter as the Gunnison Basin, in Southwest Colorado. In its 3,508 square miles, the basin extends from 7,000 feet to 14,000 feet and encompasses sagebrush, montane, sub-alpine, and alpine ecosystems (Neely et al. 2011). Climate processes vary across the basin with total average annual precipitation differing by a factor of four and a temperature range of up to 120 °F over the course of a year (https://wrcc.dri.edu/cgi-bin/cliGCStT.pl?cogunn). The Gunnison Basin is 85% public lands, including 51% Forest Service, 24% Bureau of Land Management, 8% state and local, and 2% National Park Service, and many local livelihoods rely on public lands. In two of the three counties, 12% of jobs are with the Forest Service (Cheng 2006). Basin-wide, agriculture, primarily ranching, accounts for 10% of employment, with livestock grazing occurring on the vast majority of private (96%) and Forest Service (89%) lands (Cheng 2006). In addition, tourism provides nearly 23% of the jobs (Department of Local Affairs 2010a; 2010b). Further, previous research in the Gunnison Basin found that residents had deep understandings of local weather and climate (Clifford and Travis 2018) and species-specific knowledge useful to conservation efforts (Knapp et al. 2013).

The Gunnison Climate Working Group (GCWG) is a public–private partnership consisting of agency staff, private landowners, local government, academics, and non-governmental organizations that have been working together to prepare for climate change since 2009. In 2011, the GCWG, in collaboration with The Nature Conservancy, completed vulnerability assessments for ecological systems and species of interest (Neely et al. 2011) and human communities (Knapp 2011). The Gunnison Basin is similar to many landscapes in the Intermountain West with its dominance of public lands and ranching, alongside tourism and second homes, but the GCWG and climate adaptation planning make it distinct from other communities in the region where this type of planning is in more nascent stages.

### The Southwest Colorado Social-Ecological-Climate Resilience (SECR) Project

The research presented here was part of the Southwest Colorado Social-Ecological-Climate Resilience (SECR) Project. SECR is an interdisciplinary, multisite, multi-institutional project working to facilitate climate change adaptation that contributes to social-ecological resilience, ecosystem and species conservation, and sustainable human communities in southwestern Colorado. The SECR team includes social, biological, and climate scientists as well as non-governmental organizations who work in collaboration with public land management agencies and the GCWG. In the Gunnison Basin, SECR activities focused specifically on sagebrush and spruce-fir landscapes, which were selected by the GCWG based on their social and ecological importance as well as their vulnerability to climate change. The SECR project included social and biophysical research and participatory processes that integrated scientific knowledge, land management decision-making, and local needs. In addition to the research described here, the project included ecological vulnerability assessments, social-ecological response models, and institutional analysis, and culminated in stakeholder workshops to identify specific adaptation strategies for sagebrush and spruce-fir landscapes.

### Narrative Climate Scenarios at the Landscape Scale

Climatologists and ecologists on the SECR team built the narrative climate scenarios at the landscape scale (referred to here as the Gunnison scenarios) from model data and existing research on climate impacts to the target systems for use in the focus groups. The Gunnison scenarios were designed to represent a range of plausible futures for the Gunnison Basin and thus explicitly account for uncertainty regarding the local hydrological and ecological impacts of climate change. Similar scenarios have been used by the National Park Service (National Park Service 2013) and elsewhere in Montana and Colorado (Wyborn et al. 2014, Murphy et al. 2017). Seventy-two global climate models and two greenhouse gas emissions scenarios (8.5 and 4.5 RCPs-Representative Concentration Pathways) from the Coupled Model Intercomparison Project—Phase 5 (CMIP5) (Taylor et al. 2012) were used to identify three climate scenarios for the Gunnison Basin for 2035. These scenarios included (i) a hotter, drier future; (ii) a warmer future with increased annual precipitation; and (iii) a future with high interannual variability in climate with hot, dry years and warm, wet years occurring in unpredictable cycles. The scenarios were titled Hot and Dry, Warm and Wet, and Feast and Famine, respectively (see Table 2 and Appendix 1).

**Table 2:** Summary of Gunnison scenarios: three climate scenarios for the Gunnison Basin for 2035, (See Appendix 1 for full scenario text)

Scenario title	Climate description
Hot and Dry	Sustained and long-duration drought conditions; chronic dry conditions in summer accompanied by heat waves
Warm and Wet	Water availability does not change but climate is warmer driving more rain than snow, earlier snowmelt, and a longer growing season
Feast or Famine	High interannual climate variability with hot, dry years followed by warm, wet years, driving more intermittent floods and droughts

Gunnison scenarios

Next, experts in local ecology and a literature review of climate niches for dominant plant species described how key features and systems of the landscape would change in response to new climate patterns. This information was used to connect the climate scenarios with possible landscape-scale changes. Scenarios were detailed, accessible, and provided in narrative format with quantitative information where appropriate (Rondeau et al. 2017b; 2017c). They also included climate analogs (e.g., by 2035 Gunnison will have the current climate of Saguache under a Hot and Dry scenario) and comparisons to past events (e.g., roughly every fifth year, conditions will be similar to the extreme drought in 2002) to make future change more tangible (Dunn et al. 2015). The 20-year timeframe was used because it is a timescale that is often preferred by natural resource managers for planning (Murphy et al. 2016). Draft scenarios were reviewed by scientists with relevant expertise from outside the SECR project and revised based on their recommendations.

### Interviews and Focus Groups

We utilized in-depth semi-structured interviews and focus groups to gain insight into agency decision-making and management approaches specifically as they related to climate adaptation. Both interviews (*n* = 20) and focus groups (*n* = 18)^1^, draw primarily on the same group of engaged managers, including but not limited to members of the GCWG, made up of staff from the Forest Service, Bureau of Land Management, National Park Service, Colorado State Forest Service, Colorado Parks and Wildlife, and Natural Resources Conservation Service (see Appendix 2 for a breakdown of participants by agency). All focus group participants were state and federal land managers. We focused specifically on land managers since most of the Basin’s land, especially the higher elevations, is publicly owned and managed, and because managers are tasked with planning for climate change. Participants included experts in forestry, wildlife biology, range management, botany, fire management, and hydrology. In addition, participants ranged from line-officers to specialists. Focus groups intentionally mixed participants so that each group had representatives from different agencies and disciplines. Each focus group included individuals from different agencies to better understand the views and practices of land managers across agencies and allow for dialogue across different institutional contexts. Three of the focus groups were conducted in Gunnison; one was held in Montrose.

An interview guide was used in both interviews and focus groups to ensure comparability while providing flexibility for participants to raise unanticipated topics (Patterson and Williams 2002). Probes were utilized to explore specific responses in more depth.

In the interviews, participants were asked questions about current climate impacts, future conditions, management approaches, capacity to respond, and decision-making in the face of uncertainty. In the focus groups, participants read through each scenario and then answered a series of questions about impacts to local resources and livelihoods, potential responses and barriers to responses, opportunities, conflicts, collective action, and decisions in the context of change and uncertainty. At the end of the focus groups, participants were asked for feedback on the utility of scenario process (see Appendices 3 and 4 for interview and focus group guides). The interviews and focus groups were designed to serve as a standalone social science research project as well as to inform subsequent stages of the larger SECR project. Interviews lasted ~1 h and focus groups lasted ~2 h. Both were audio-recorded and transcribed verbatim. Transcripts were coded in NVivo^2^ using both descriptive (water resources, barriers, etc.) and interpretive (risk, fear, frustration, etc.) codes (Richards 2005) (see Appendix 5 for codes).

An iterative analytical process was employed to compare empirical findings with theoretical concepts from the literature (Layder 1998). This iterative process compared existing research and frameworks on relevant topics, such as resilience, transformation, risk, and institutional culture, with interview data to produce an understanding of how interview results aligned with and/or extended current theory. To do so, we re-reviewed relevant literature at multiple points during the analysis (e.g., while building the initial coding framework, before coding individual interviews, before comparing across interviews, before writing the results section of this paper) and then foregrounded that literature in the next stage of analysis. While existing theory informed coding, the codes were not solely predetermined based on the literature. We used a modified grounded theory approach to attend to emergent findings, with many codes drawn from the interview data in addition to the a priori codes. This iterative process enabled us to systematically examine interview data in light of existing research while also allowing for new, emergent findings. The excerpts below represent views that were common across the sample (except where explicitly noted).

## Results

### The Past, the Future, and the Question of Transformation

The interviews and focus groups were designed to engage managers in thinking about how they would respond to a range of local climate change impacts. Below we examine the ways that managers conceptualize future change and the management approaches they support. Some of the differences described below hinge on whether managers were looking to the past to guide their response to climate change, relying on historic ecosystem states and tried-and-true management approaches, versus looking to the future toward novel ecosystems and innovative strategies.

Managers looked to the past for guidance in the context of climate change in two main ways. Some managers focused on resistance, the goal of retaining historic ecosystem types in their current locations, as a means to protect vulnerable species or ecosystem features from the impacts of climate change. Exemplifying this view, one manager suggested that resistance was a valuable tool in the context of adaptation, saying “we need all the tools in the toolbox…in order to respond and to keep a forest there or to keep sagebrush land there. I mean you need to be able to do human intervention”. For this manager, active intervention is required to retain an ecosystem from the past in a specific location, given the impacts of climate change.

Managers also looked to the past by comparing climate change to previous disturbances. These managers argued that since climate change was just another disturbance, business as usual would be effective. As one manager shared, “we expect to deal with disturbances and we have a ton of experience dealing with disturbances. Climate change is just one more disturbance. That is how we view it”. For this manager, experience with past disturbance created a confidence that existing management strategies would be effective in the context of climate change.

In contrast, other managers had their eyes clearly on the future, embracing ecosystem change, including regime shifts, and trying to envision new approaches. Breaking with decades of federal land management largely focused on managing for historic ecosystem states, many managers in this study discussed the need to actively assist ecosystems in transitioning to new states. They believed that climate change impacts would be too dramatic to effectively resist and that regime shifts would be inevitable. One manager explained that “If there’s a disturbance there, we wouldn’t go back in and try and recreate that. Let’s adapt it to what it’s moving towards…If it needs adaptation there, let’s not try to get spruce-fir to grow again. We’re going to fail”. According to this manager, in the context of climate change, resistance is futile. For those who saw regime shifts as inevitable, active intervention was often the preferred approach. This manager described this approach, saying: “We’re trying to adapt hands-on, adapt that system so that it’s either more resilient or anticipate what’s going to happen and get it in front of it, maybe steer what may end up happening as a result of climate change”.

Active intervention was synonymous with assisted migration for many managers. In response to projected changes to the historic range of ponderosa pine, one manager suggested “I would see that as more [of] …us artificially migrating that species, because ponderosa pine… the seed doesn’t move very far”. They recognized that ponderosa pine would not easily adapt without management intervention, in this case assisted migration. Seeding outside of historic ranges was often recommended as a transformation strategy to assist with regime shifts and species migration. Another manager suggested mixing in Douglas fir seeds when replanting areas that are currently spruce-fir. A third manager argued that “you’ve got to start gathering seeds and planting limber pine at 11,000 feet”. In short, these managers recommended that management actions aim for future ecosystem conditions, presumably using downscaled climate projections, and that managers actively intervene to guide ecosystem change toward those future conditions.

But other managers were less certain about active intervention, arguing that it was difficult to determine which approach would be best. As one manager asked:

Should we be bumping up an elevation to the next seed zone in order to have trees that would be a little bit more well-adjusted to a warmer climate? Especially when you’re dealing with stands that have a 300-year rotation on them, it’s difficult to try and figure out whether it’s an appropriate action.

For this manager, the long rotation of spruce-fir stands called into question projections about how those stands might change over the next few decades. Another manager described a similar struggle, saying: “I’m in a philosophical quandary right now…the two schools of thought, the new thinking: maybe we need to be more manipulative and get in front of this. And the other that says: what’s wrong with letting it play out and seeing where things go.” In calling it a “philosophical quandary”, this manager implies that the question is, in part, moral or ethical, rather than just scientific. Thus, for some managers, opposition to transformation was grounded in a lack of confidence in climate projections. But for others, it was a philosophical question of what constitutes appropriate management. This manager weighs the risks of transformation versus waiting and watching, saying:

You don’t know what the situation is so you’re going to try to manipulate the landscape in ten different ways in case it hits any direction so you’re going to have at least nine failures and nine damaged creations, if you will. So yeah, that’s a really bad idea in my opinion because the ecosystems that are around are for a long time, and if we do nothing it will come back to some kind of homeostasis and without us it may not be the way we like and we may lose some species locally or worldwide…but it’d be probably better off than us trying to manipulate.

For this manager, uncertainty about future conditions meant that active intervention could cause harm or damage to ecosystems. They weighed the possible harms from active intervention against the possible harms from climate change (e.g., local extinctions) and determined that the former was more problematic. They also expressed confidence that ecosystems would achieve some sort of homeostasis (i.e., equilibrium) over time, despite climate change. Another manager agreed, saying:

I don’t know that we can adequately predict any changes in range of spruce-fir or anything else. I think we should be able to expect things to behave different as we go in…I don’t think we should plan to relocate or replant spruce because I think it’s such a long process that spruce is going to do its thing on its own at some point.

Again, a lack of accurate predictions about future ecosystem conditions meant that assisted migration is inappropriate, for this manager. They support an active timber program and the kind of stand-level intervention involved in timber management. Thus, they are not expressing opposition to active management, but rather opposition to transformation to new ecosystem or vegetation types projected to be more adaptive under future climates. Here another manager argues in favor of active management, but against transformation, saying:

Just to let certain things happen and try to adapt your expectations, and maybe the movement up of sagebrush and the conversion in the lower elevations to grass might actually support [grazing] there, as long as you keep the cheatgrass out.

This manager also accepts that ecosystems will change, but similarly argues that managers should not pursue transformation. At the same time, they support grazing and weed management, so they are not advocating for a purely hands-off approach.

Interestingly, some managers saw resilience as a middle path whereby active intervention could assist ecosystems in adapting to change without presupposing specific future conditions or ecosystem type. As this manager argues:

Recognizing that there is change afoot and that the more resilient we can make the spruce-fir type, and any of our other forest types, to create an insurance that whatever happens that we have a more resilient stand out there that can take of what’s ahead of it…I am not anticipating anybody coming up with a magic bullet that says on that particular acre it’s going to be like this, this is the condition that is going… 500 years from now. There is no way for us to do that…we have to create a landscape that deals with whatever conditions. And recognizing that spruce-fir may move up the hill, and ponderosa pine may move in behind it. Or vice versa. We don’t know. The more resilient the landscape we make, the more it will be able to adapt.

Interestingly, they are advocating for active intervention to increase adaptability and future resilience, not to guide ecosystems toward specific future conditions. While these recommendations were vague in the sense that they did not specify how resilience will be increased, they do point to a middle path where managers actively intervene to assist with adaptation without presuming to know specifically how ecosystems will transition. Another manager described resilience strategies similarly, explaining that “you need to find what’s an appropriate middle ground and try to sustain that, adapting for bad years and good years”. This approach requires acknowledging and accepting that future ecosystems will be different from past ecosystems, but does not require relying on specific projections about future ecosystem conditions. In this sense, resilience is seen as a way to minimize risk and hedge in the context of uncertainty.

### Scalar Challenges and Institutional Constraints to Effective Adaptation

The question of how to manage public lands in the context of climate change often evoked discussions about temporal and spatial scale. In interviews and focus groups, managers grappled with both the scale of projected climate impacts and their inability to work at larger scales. As one manager put it, “I think probably the biggest challenge we face is scale”. Below we examine the scalar challenges that managers discussed, including specific scalar mismatches and the institutional constraints that influenced the scale of management action.

Some managers advocated for large-scale management intervention because the scale of climate change impacts demanded a large-scale response. These managers focused on projected climate impacts, from more intense drought and wildfire to expanding forest pathogens, arguing that none of these could be addressed at a small scale. As in the section above, managers here acknowledged that the future would be different from the past, suggesting that disturbance and ecological change would happen at a larger scale. As a result, they concluded that small-scale responses to large-scale ecosystem changes were insufficient to address climate change. This manager articulated this view, arguing for management interventions that matched the scale of the change:

I feel like we don’t address the right scale either… We’re seeing these large-scale responses [referring to the scale of pine beetle outbreaks] and to think that we can actively manage it at a scale other than a large-scale sort of misses the point. I think the management response is going to have to match the natural response spatially and temporally.

Managers conveyed that they were hearing “a lot nationally from agency leadership about increasing the scope and scale of landscape restoration”. But many managers felt frustrated about their inability to work at the required scale. This manager described a small-scale project to build resilience, but concluded that it may not be having an impact at the scale needed, saying:

We can’t make it get colder so the tree can continue to survive but did what we could. We opened up the canopy and got rid of the competition for sunlight and then we came back in over the next few years and if there were four trees here you picked the healthiest one and cut the other three and leave the one. We were actively managing those small groves of trees to try and keep those species growing there. So you can do that, but it’s such a small-scale thing that it’s like you’re taking care of one small part of a thousand-mile beach. And it’s frustrating because it’s hard to feel like you’re accomplishing much sometimes.

This manager recognized the need for small-scale interventions to influence larger scales, as theorized by panarchy, but worried that this particular intervention might not create resilience at the landscape scale. Many managers expressed concerns about the scope of the problem, and their inability to address it at scale. This manager sums up that perspective.

Just the scope and scale of the impacts…whether it would be the spruce bark beetle or the mountain pine beetle or cheatgrass or those things that are on such a large-scale …They’re too big to deal with, to be able to really make drastic effects. You might be able to do a little thing here or a little thing there, but the grand scale of these impacts are pretty big.

The mismatch between the scale of management action and the scale of climate impacts was a frequent source of frustration.

For many managers, specific institutional constraints limited the ability of public land management agencies to implement large-scale strategies. As this manager put it, “a lot of it has to do with the amount of landscape that we’re actually able to manage is very small”. One manager lamented that the Forest Service was bound by management rules and constrained by limited resources such that “we work at a scale where we couldn’t even stop [climate change impacts] if we wanted to. For most of this we don’t have a choice”. Some managers connected these constraints to different management approaches (e.g., resistance, resilience, and transformation), arguing that resisting change would simply be too expensive given the large landscapes that agencies manage. In the excerpt below, this manager suggests that resistance is not feasible, saying:

[Resistance] in certain places is going to be basically fruitless or so expensive that you need to look at what is it going to become, and just let it do that. Let it convert. If we’re going to have sagebrush move up a 1000 feet, then let it do that and maybe encourage that, and maybe try to encourage the grasslands down below from becoming cheatgrass.

There is an important tension between the desire to respond at a scale that aligns with the scale of ecosystem change, and practical institutional constraints to marshaling action at that scale.

In addition to a lack of resources to intervene at large scales, managers also discussed policy requirements and lack of public support as important constraints. According to this manager:

Spruce-fir type, from a long-term landscape approach, doesn’t really do anything in small pieces. It’s a lot like lodgepole, when it recycles itself, resets, it does so in really large scales. It doesn’t do it in five-acre patches. Huge disturbances. That scale is hard for people to get their brains around. Both from a regulatory standpoint in terms of what we do or don’t do, where we have the availability to actively manage, but also from a social acceptability standpoint, folks don’t like to see 10’s to 100’s of thousands of acres of their national forest reset all at once.

Here again, the governance context, specifically policy and public support, is influencing the ability of managers to work at specific scales.

### Innovative Ways to Nest Different Approaches Across Scales

Some managers solved the scalar dilemma by nesting goals and approaches across scales, pursuing smaller-scale strategies intended to influence or add up at a landscape scale, as described by panarchy. Looking at these nested approaches provides insights into how ideas about the past and the future connect to different management approaches (e.g., resistance, resilience, and transformation) and to concerns about the uncertainty of projections about future ecosystem conditions.

For example, actively managed “islands” were envisioned as a way to work at multiple scales, utilizing a combination of active intervention and hands-off management. Managers advocated for intensive management of smaller patches or islands that could provide habitat for key species or ecosystem types in the context of climate change. One manager described this perspective, saying:

I suspect we’ll have islands of spruce-fir and we can manage those islands…to try and maintain those disturbances, maintain that genetic diversity so that it’s the locations where these are faded out we’ll regenerate spruce-fir, that population then can re-establish itself. But I think the days of large thousands and thousands of acres, of contiguous spruce-fir, are for the short future, so you won’t have to worry about managing those, they’ll be something different. We’ll have to figure out what that “different” is and if it will eventually shift back to spruce-fir, which will take quite some time.

They focused on how to manage spruce-fir islands, essentially a relic of the past, at a small scale in ways that enable future shifts back to widespread spruce-fir ecosystems. Thus, resistance at smaller scales can maintain past patterns of disturbance and existing genetic diversity to preserve future options across the landscape. This strategy enables managers to employ resource-intensive, resistance strategies in select locations to preserve future options and build resilience at a much larger scale. In another example, managers suggested improving water infiltration and storage to enable water-dependent species to persist in their historic ranges even as precipitation decreased. Again, resisting ecological change at the scale of these patches or islands was envisioned as an active, targeted, smaller-scale strategy to retain the values of past ecosystems and increase resilience at a larger scale, both now and into the future. These islands resemble refugia in many ways, but are clearly seen as small-scale and managed through active interventions that maintain historic ecosystem characteristics (in contrast to refugia that are larger-scale and maintain historic conditions without active intervention).

Thinking about combining small-scale interventions was also seen as a way to start working at a scale aligned with the impacts of climate change. According to this manager, these small-scale interventions could add up to larger-scale benefits:

My point is every little thing we do is important, it has a cumulative effect, it makes a difference for that drainage and that piece of forest and so on and we need to be doing those things. But it’s really discouraging when you look at the whole deal.

This excerpt illustrates the tension between trying to have an impact at scale, albeit through smaller actions that add up, and overwhelming scale of the problem. Interestingly, some managers suggested that, while a single agency might not have the resources to work at larger scales, agencies could combine resources to work across jurisdictional boundaries to coordinate and implement actions that have an impact at larger spatial scales. For example, one manager said that under future scenarios “there is going to have to be even more where agencies are working together to accomplish things, so having a cross-boundary strategy” was important as opposed to operating independently.

Managers also explained that smaller-scale experimentation would build knowledge necessary to expand interventions to larger scales. As this manager put it:

Since we’re doing a lot of stuff on small scale, private land, limited budgets, we feel pretty comfortable. I feel pretty comfortable, we do a lot of trial stuff, whether there’s a question of this will work, okay, instead of doing this across the whole 640, let’s do a low 30-acre patch. How does that work, what can we do differently? So a lot of those adaptive management trial and error, we’ve learned a lot from our mistakes.

This manager felt more comfortable with the small-scale interventions, which is how they resolved the dilemma of doing active intervention on a large-scale with imperfect knowledge of future conditions. Another manager suggested something similar, saying:

The other concept that comes to mind is that of experimentation. Maybe you don’t do everything everywhere. Maybe you try something on BLM land. Maybe it works, maybe it doesn’t…a series of experiments around the Basin for example and you try to watch and see which ones work best and you try and emulate that in different places.

Again, these managers solve some of the challenges related to scale and transformation by nesting their approaches spatially and temporally. Initial experiments at smaller scales were characterized as providing knowledge and support for future interventions at larger scales.

## Discussion

This study set out to learn how managers envision future ecosystem change, and how they view different management approaches in the context of climate adaptation. Some managers in this study emphasized the past, arguing that climate change impacts were similar to previous disturbances and relying on existing management approaches. Other managers framed climate adaptation in terms of a novel future, arguing for transformation, and suggesting management actions to assist and even guide ecosystems toward future states. But characterizing adaptation as either looking backward to the past or forward to the future maybe an oversimplification. Murphy et al. (2017) found that many people drew on past experiences to help them conceptualize future impacts and potential responses. Dilling et al. (2017) agree, arguing that while the past is not always an accurate representation of the future, it can provide useful insights into both the drivers of particular problems and the suite of potential solutions. Some adaptation efforts intentionally utilize analogs from the past to facilitate decisions about the future (Ford et al. 2010; Glantz 1991). The past is often used, then, to fill in the gaps when our knowledge of the future is limited.

However, if we envision future change as different from past change, looking to the past could be problematic. Kareiva and Fuller (2016) argue that responses to climate change are “too often business as usual” due to an overemphasis on historical reference points. In this study managers who argued that future climate impacts would be similar to past disturbance also claimed that conventional, tried-and-true management approaches would work. Opposition to transformation was grounded in the claim that we have imperfect knowledge of future climates and what kinds of ecosystems will be adapted to those climates. However, in the context of imperfect knowledge, Kareiva and Fuller (2016) recommend that managers emphasize innovation and experimentation, even if some actions seem risky. They argue that all courses of action (including no action) will result in harm. The expectation is that many experiments will fail, but that some will succeed and provide important knowledge for adaptation. To account for this, Joyce et al. (2008) recommend that agencies adopt *safe to fail* policies to promote just this kind of experimentation.

In this study, ideas about the effectiveness of different approaches were very much linked to scale. To date, much of the research on scale and climate adaptation has focused on scalar mismatches between climate information and decision-making (i.e., the spatial scale is too large and/or the temporal scale too long to be useful to decision-makers) (Mase and Prokopy 2014). Research on scale has also examined the cross-scalar interactions that produce specific vulnerabilities (Leichenko and O’Brien 2008). Some researchers have suggested that smaller-scale interventions might accumulate to promote resilience or transformation at larger scales, drawing on the concept of panarchy to illustrate cross-scale interactions (Chaffin et al. 2016, Folke et al. 2010, Gunderson et al. 2017). Our study highlights the important ways that scale impacts climate adaptation, specifically the key role scale plays in the selection of management approaches (e.g., resistance, resilience, or transformation) and the question of intervention (e.g., hands-off or active intervention). To the extent that climate change was seen as driving large-scale ecological change, managers in this study worried that their responses would not match the scale of the impacts anticipated under climate change and therefore be ineffective.

A range of institutional constraints, from insufficient budgets to small staff, and from regulations to lack of public support, were cited as reasons that agencies were unable to work at large scales. These are similar barriers other studies have identified for climate adaptation in land management contexts (Archie 2014; Hagerman 2016). More specifically, many managers considered resistance impractical due to both institutional and biophysical constraints because of the kind of ongoing intervention required to maintain past ecosystem types in the context of future climates. At the same time, some managers expressed concerns about the efficacy of large-scale interventions given imperfect information about future climates and ecosystems. In sum, scalar challenges and mismatches left many managers in a quandary regarding how to respond to climate change, reflecting the same dilemmas other scholars describe (Cole and Yung 2010).

One of the ways that managers resolved these scalar dilemmas was by nesting approaches both spatially and temporally. Managers suggested that small-scale patches could be actively managed to retain past ecosystem features and provide for resilience at larger scales through maintaining the genetic diversity required for future ecosystem change. In addition, small-scale interventions could provide opportunities for learning about the efficacy of different treatments before implementation at larger scales. During stakeholder workshops that happened later in the SECR project, managers also discussed the possibility of combining resistance and transformation. For example, in order to protect habitat for Gunnison sage-grouse, they envisioned resisting juniper invasion of sagebrush landscapes in the short term while assisting as higher elevation areas shift into sagebrush habitat, and then transitioning away from active intervention once adequate habitat existed at those higher elevations (Rondeau et al. 2017b).

The innovative strategies designed to nest smaller interventions within larger landscape scale goals demonstrate the ways that managers are negotiating the scalar challenges of adaptation and the institutional constraints that they face, and how they envision cross-scale, social-ecological interactions, which aligns with the literature on panarchy (Chaffin et al. 2016; Folke et al. 2010; Gunderson et al. 2017). Further, these strategies enable managers to simultaneously focus on the past (e.g., through small-scale patches where resistance is employed) and the future (e.g., through large-scale ecosystem transformation). Nested approaches also helped resolve some of the uncertainty about future ecosystem conditions and the discomfort some managers felt about transformation. Small-scale interventions were necessary in the context of institutional constraints, but they also provided the opportunity to experiment and learn before scaling up. However, this temporal nesting, small-scale now and larger-scale later, assumed that tensions related to the both institutional constraints and the scale of transformation would be resolved in the future.

In addition to nested approaches, resilience was seen as a way to resolve some of the dilemmas described above. Some managers argued for active intervention to promote resilience, suggesting that resilient ecosystems would have the capacity to adapt to a range of future climatic conditions. In this sense, resilience offered a middle path, enabling managers to assist ecosystems in adapting to future conditions without predicting specific future ecosystem states. However, it was not entirely clear what specific management actions these managers wanted to employ to increase resilience. The vagueness of these statements about resilience is consistent with previous research that found that while the ambiguity of resilience makes it difficult for managers to operationalize, it also provides flexibility that some managers value (Timberlake and Schultz 2017). Interestingly, during subsequent SECR meetings, managers and the research team co-produced a suite of specific resilience strategies, including restoring degraded meadows for hydrologic recharge, creating forest mosaics, and retrofitting climate-wise culvert replacements, indicating that managers in the area have some capacity to connect the concept of resilience with specific management actions (Rondeau et al. 2017b; 2017c).

In this study, while some managers resisted transformation, questioning projections about future ecosystem states and arguing that untested approaches would be too risky, most managers readily envisioned future ecosystems as different from past ecosystems, acknowledging the potential for regime shifts (even if they reached different conclusions regarding appropriate management actions in the face of these shifts). This future orientation might have been inspired by the scenarios that were utilized in the focus groups, since they forced managers to think about specific plausible futures for the Gunnison Basin. However, similar scenario processes in Colorado found that most participants were reactive and incremental, as opposed to proactive and transformative (Wyborn et al. 2014). Thus, managers in the Gunnison Basin appeared to have more capacity to embrace future ecosystem change and consider long temporal scales, as compared with participants in similar research projects elsewhere. At the same time, during a SECR stakeholder meeting that occurred after the interviews and focus groups, managers suggested that transformation was particularly challenging because of lack of adequate information to determine which sites were transitioning to new states (Rondeau et al. 2017b; 2017c).

## Conclusion

With greater attention to climate adaptation on public lands, it is critical to understand how managers envision responding to local impacts. In this study, perceptions of environmental change shaped how managers responded to climate change, at what scale (patch or landscape), with which objective (resist or transform), and drawing on what tools (existing or novel management approaches). Our findings help explain why climate adaptation varies according to ecological and institutional context, contributing to a growing body of research on how climate adaptation is implemented on public lands.

Adaptation is inherently political (Pelling 2010; Eriksen et al. 2015; O’Brien and Selboe 2015), involving numerous trade-offs and near-certain conflict. But in these interviews and focus groups, managers framed decisions about how to respond to climate change primarily in ecological terms. However, it is important to note that framing decisions about climate change adaptation solely in terms of ecological conditions obscures the political nature of these decisions (Eriksen et al. 2015). And while transformation is primarily discussed in this paper with regard to regime shifts or disruptions to ecosystems, this discussion also raises questions about another kind of transformation, the transformation of institutional structures and cultures (as discussed by O’Brien and Sygna 2013). Responding effectively to the ecological changes wrought by climate change requires social transformations that enable institutions to become more nimble, support risk-taking and experimentation (while also ensuring accountability to the public), navigate uncertainty and trade-offs, and work across varied temporal and spatial scales. How public land management agencies can embrace transformations of ecosystems and corresponding institutional transformations is an important question, one that highlights the social-ecological linkages that permeate climate change adaptation. An improved understanding of how land managers approach climate adaptation and the challenges they face as they respond to change can also provide insights into the policy and institutional changes that can support these decisions.

## Supplementary information

Appendix 2

Appendix 1

Appendix 3

Appendix 4

Appendix 5

## Footnotes

1 The 18 participants were split among four focus groups, specifically in groups of 5, 3, 4, and 6 participants.

2 https://www.qsrinternational.com/nvivo-qualitative-data-analysis-software/home
