# Supplementary material for: Navigating Climate Adaptation on Public Lands: How Views on Ecosystem Change and Scale Interact with Management Approaches
Source: Environ Manage. 2020 Jul 29;66(4):614–28. doi: 10.1007/s00267-020-01336-y (PMC7522104; doi:10.1007/s00267-020-01336-y)
Supplement: Supplementary file 1 — Appendix 2 [file 267_2020_1336_MOESM1_ESM.pdf]

Appendix 2: Number of Interview and Focus Group Participants from Each Agency.

Agency	Interview Participants	Focus Group Participants
Bureau of Land Management (BLM)	7	5
U.S. Forest Service (USFS)	7	6
Natural Resource Conservation Service (NRCS)	2	1
National Park Service (NPS)	2	3
State Agencies (forestry and wildlife)	2	3
