# Supplementary material for: Navigating Climate Adaptation on Public Lands: How Views on Ecosystem Change and Scale Interact with Management Approaches
Source: Environ Manage. 2020 Jul 29;66(4):614–28. doi: 10.1007/s00267-020-01336-y (PMC7522104; doi:10.1007/s00267-020-01336-y)
Supplement: Supplementary file 2 — Appendix 1 [file 267_2020_1336_MOESM2_ESM.pdf]

Appendix 1: Narrative Scenarios

A team of ecologists and climate scientists developed narrative climate scenarios to translate climate projections into descriptions of local terrestrial changes for the Gunnison Basin. Three scenarios were developed to encompass a range of future conditions for the Gunnison Basin in the year 2035 (a 20-year projection). These scenarios were used in focus groups with resource managers to guide discussions of how they would create management strategies in response to the changes described.

Scenario 1: Hot and Dry

In this scenario annual temperature increases approximately 5^0 F by 2035. To put that in perspective, Gunnison's temperature becomes similar to the current climate of Ridgeway, CO. By 2035, every summer will be warmer than 2002 and 2012 – years when we experienced excessive heat waves. At elevations below 7,000 feet, for at least two weeks during the summer, nighttime lows will not dip below 68^0 F (a typical tropical night), and summer will expand by a month. **Annual precipitation will decline by 10%, and the combined effect of warming and lower precipitation will result in nearly 45% decrease in annual runoff. There will be a large increase in the frequency of extreme drought years.** Roughly every fifth year, we experience droughts similar to 2002 and 2012 (in these years, precipitation was 40% below average).

FIRE

Not every year will be an exceptional fire season but average fire frequency, intensity, and size will increase. The average fire season will lengthen by one month and the average fire frequency will increase up to 12 times while the total area burned in any given year will increase 16 times[1]. The largest burns will be in coniferous forests, including spruce-fir, lodgepole pine, mixed-conifer, and ponderosa pine. Once burned, these areas are likely to transform into aspen, shrublands, or grasslands. The growing season will increase by three weeks, however, with less precipitation the understory herbaceous growth (fine fuels) will decrease which may reduce fire risk in the sagebrush. If a fire occurs in the lower elevation sagebrush zone the site will transform into grassland or rabbitbrush/grassland rather than return to a sagebrush

system. There is a good chance that the “new” grassland will be dominated by cheatgrass. Note that sagebrush requires at least 7.5 inches of annual precipitation, and the large water stress in this scenario will make it difficult for the low elevation sagebrush to regenerate.

DROUGHT

In this scenario, Gunnison’s annual precipitation declines and becomes similar to the current precipitation of Del Norte[2]. Spring snowpack will decline by 10% and spring temperatures will increase by 4^0 F. This combination of a reduced snowpack and warmer spring temperatures will reduce the available water during the growing season. Trees and shrubs (especially sagebrush) rely on winter and spring snows. The snowpack allows for deep soils to remain moist during the growing season, therefore a reduced snowpack associated with a warmer and drier spring will negatively impact vegetation with deep roots (most trees and shrubs). Summer precipitation will decrease by 20% and have a large negative impact on vegetation, especially shallow rooted plants (mostly grasses and forbs). Snowline shifts up by 1200 feet and could impact the lower elevations of the Crested Butte ski resort. In addition, the average timing of snowmelt will shift a full three weeks earlier from temperature increases and more frequent dust-on-snow events (which will occur every year). Higher than average peak spring flows followed by lower summer flows will reduce the amount of water available for fish, riparian vegetation, migratory birds, and grazing animals, especially during summer. Endangered fish would most likely suffer from lower in-stream flow and increased stream temperature. Less precipitation in winter and summer will significantly decrease surface water and shallow ground water. Seeps, springs, and mesic meadows associated with shallow groundwater will decline and species composition will be greatly altered. We will likely see a shrub invasion into mesic meadows and a decline in nearby aspen stands.

INSECTS

Tree mortality due to insect and disease outbreaks will greatly increase with a hot and dry climate, more so than in any other scenario. The current spruce-bark beetle infestation will likely expand and cause significant mortality in the mature trees[3].

Species that rely on mature spruce-fir forests, such as Lynx, Boreal owl, Snowshoe hare, and Pine marten, will decline due to lack of food and shelter. Aspen trees at lower elevations will experience die-back associated with increased temperatures and decreased soil moisture. However, aspen stands at upper elevations may increase as coniferous trees decline due to fire and beetle kill.

Scenario 2: Warm and Wet

In this scenario, annual temperature increases 2^0 F by 2035. To put this in perspective, temperatures in Gunnison will resemble current temperatures in Cimarron. **Summer will expand by a week. Annual precipitation will increase by 10%** (in terms of soil moisture and stream flows a 5% increase in precipitation is needed to offset a 2^0 F increase in temperature with its associated higher rate of evapotranspiration). Drought years, such as 2002, will occur every 15th year, similar to today's frequency. However, the intensity and severity of droughts will increase because of higher temperatures.

CHANGE

While the water stress from 2^0 F temperature increase will be offset by a 10% increase in precipitation, ecosystems will change in measurable ways. For example, the ratio of warm season to cool season grasses will change, and we could see declines in western wheat grass, needle and thread grass, while blue grama and galleta grass expand. The snowline will shift upwards by 600 feet. As a result, the current vegetation in the 8,500-9,000 feet elevation band will begin to shift from mixed conifer or aspen to ponderosa pine. Due to increased precipitation, overall runoff will increase by 10%, while warmer temperatures mean that peak runoff will occur a week earlier. In this scenario, heat waves similar to 2002 (5^0 F above normal) will occur once every decade. Fire risk in this scenario is the lowest of any scenario but fires will be present, and intermittent dry conditions may cause severe fire hazards because of high fuel loads. These high fuel loads are a result of increased winter, spring, and summer precipitation producing more foliage. A 2^0 F increase in temperature will increase the fire frequency up to 4 times and the annual area burned by 6 times[1].

WEEDS

We will have greater than normal winter snowpack above 10,000 feet and spring, summer, and fall precipitation will increase at all elevations. The increase in year-round moisture coupled with a moderate increase in temperature will promote invasive species (more so than any other scenario). Current invasive species such as leafy spurge, knapweed, and yellow toadflax will expand into low to montane elevations and new invasive species such as Japanese brome or purple loosestrife will likely move into the area. Rangelands will become degraded by invasives, and knapweeds and leafy spurge expand into rangelands that have never had a serious weed problem. Further, invasive species will out-compete the native vegetation and create a high density of fine fuels for fires, especially at the lower elevations.

WATER

We will still experience droughts; however, they will be less frequent than in the other scenarios. Disease and insect outbreaks are expected to be lower than the other scenarios, however, insect outbreaks will still increase, as the droughts that do occur will be more intense than the droughts experienced during the 20th century. When we do experience a beetle outbreak, the recovery time may be quicker than in the other scenarios. Seeps, springs, and other groundwater dependent wetlands will increase or experience very little change. There will be some drought years that impact low elevation wetlands, but for the most part, wetlands will benefit from the years of increased annual precipitation.   Higher elevation wetlands will do exceptionally well and possibly expand due to the greater snowpack above 10,000 feet. Higher soil moisture will likely eliminate or reduce invasive species in wetlands.

Scenario 3: Feast or Famine

In this scenario, annual temperature will increase approximately 3^0 F by 2035. To put that in perspective, Crested Butte's temperature will be similar to the current temperature of Lake City. Average annual precipitation does not change; however, we will experience **larger year to year fluctuations in precipitation, with some very wet years and some intense drought years,** as compared to our current climate. Winter precipitation will increase, but precipitation will decline in the other

seasons. When droughts occur, they will be more intense than present but generally less than two years long. Once every decade we will experience a drought similar to the 2002 and 2012 droughts (years when precipitation was 40% below average).

FEAST

The growing season will expand by 2 weeks and during wet years vegetation growth will be exceptional with trees, shrubs, and ground cover greatly increasing. The frequency of severe El Nino and La Nina events will double to an average of once every seven years. We experienced severe El Nino years in this region in 1982/83 and 1997/98 with annual precipitation at roughly 20% above average. Invasive species will do well under El Nino conditions but decline in La Nina conditions (drought years). The annual fire risk is lower in this scenario than the hot and dry scenario. Large fluctuations between wet and dry years will increase fuel growth during wet years. This means that when a fire does occur, the severity, intensity, and size could be very high, and in a bad fire year the average fire frequency will increase up to 8 times and the area burned will increase 11 times[1]. Year to year, summer monsoons will be more variable than they are currently. Large spring floods will be more likely as earlier rain on snow events will cause abrupt snowmelt. Dust-on-snow events, coupled with warmer spring temperatures, will also increase the chance of spring flooding, especially during El Nino years. The largest flooding events will generally occur from heavy monsoon precipitation. During these floods, there will be severe erosion in small streams as water runs over banks and culverts.

FAMINE

Intense droughts will more frequently follow extreme wet years. Bark beetles will expand during these drought years, causing extensive conifer mortality. The difference between this scenario and the hot and dry scenario is that multi-year droughts will be less likely in this scenario, so bark beetle dieback may not be as severe as in the hot and dry scenario. It is important to note that most conifer forests can regenerate more easily following beetle outbreaks than fires because bark beetles do not kill the young trees. However, insect kill in mature trees will diminish seed production. This reduction in seed crop will hurt the animals that rely on conifer seeds. In the event that a fire occurs after a beetle outbreak, tree regeneration is

nearly impossible due to a lack of a nearby seed source and nurse plants. The large fires associated with drought years will result in younger forests, more open structure, more early successional species, and more invasive species. Large landscape scale disturbances, such as fire and insect outbreaks, will fragment coniferous forests and negatively impact Lynx, Snowshoe hares, Pine martens, and other species that rely on large intact functioning forests, while possibly being a benefit to those species that prosper from a more open forest canopy.

Seeps, springs, and other groundwater dependent wetlands will experience a moderate decline, especially below 8,500 feet, where spring precipitation will fall as rain rather than snow. Increased evapotranspiration, driven by higher temperatures, will reduce soil moisture and streamflow. Consequently, species that can handle drier soil conditions, for example sagebrush, shrubby cinquefoil, and rabbitbrush will flourish; invasive species such as cheatgrass and knapweed will likely increase, especially at the lower elevations. Juniper establishment in the sagebrush is likely during wet years that follow a drought year.

1. Westerling, A.L., H.G. Hidalgo, D.R. Cayan, and T.W. Swetnam. 2006. Warming and earlier spring increase Western U.S. forest wildfire activity. Science 313: 940-943.
2. Rangwala 2014 pers. Com.
3. Colorado State 2013. 2013 Report on the health of Colorado's forests; caring for Colorado's forests: Today's challenges, tomorrow's opportunities.
