# Supplementary material for: Navigating Climate Adaptation on Public Lands: How Views on Ecosystem Change and Scale Interact with Management Approaches
Source: Environ Manage. 2020 Jul 29;66(4):614–28. doi: 10.1007/s00267-020-01336-y (PMC7522104; doi:10.1007/s00267-020-01336-y)
Supplement: Supplementary file 3 — Appendix 3 [file 267_2020_1336_MOESM3_ESM.pdf]

Appendix 3: Interview Guide

Thanks so much for agreeing to an interview. We really appreciate your time. This project is funded by the Department of the Interior's North Central Climate Science Center and builds on previous research conducted in the Basin and takes next steps by focusing on specific ecological systems. In short, we're working with representatives of land management agencies here in the Gunnison Basin to understand the changes occurring in particular ecosystems and how managers can adapt natural resource management to those changes.

Our ultimate goal is to identify adaptation strategies and develop a process that can be used by managers beyond the life of this project for considering climate adaptation in natural resource management. The project team including federal and state agencies, recently identified two systems as particularly important in this area. These are sagebrush and spruce-fir ecosystems. This project is focused on the importance of these systems both socially and ecologically to this region.

At this stage, I'm interviewing federal and state natural resource managers to learn more about how the agencies manage these systems in the context of change. The interview is too long if I ask questions about both systems, so we will focus on just one of the systems, based on the one you are most knowledgeable about, either the greater sagebrush or spruce-fir ecosystem. Which one would you feel most comfortable discussing today?

As noted above, interviewees were asked to select a focal social-ecological system—either sagebrush or spruce fir—resulting in nearly an even split between the two (sagebrush n=12, spruce fir n=10).

Do you have any questions before we start?

Can you describe your position and role in the agency? *Note: [Target] below refers to either greater sagebrush or spruce-fir ecosystems.*

Importance and Change

Can you tell me about the importance of [Target] in this area? Who uses or values [Target}? In what ways?

What do you or your agency (for tribes I wouldn't insert this phrase) hope to achieve in your management of [Target] on FS/BLM/NPS/tribal lands?

What are some of the challenges or threats you're facing in your management of [Target]? Socially, ecologically, within the organization or pressure from outside?

Based on your experience, does your agency have the capacity to realize their goals for [Target]?

In what ways do current laws and policies shape your management of [Target]

Who is impacted by changes to [Target]? (and in what ways)

Are there organizations or individuals in the Gunnison that are interested or involved in the

management of [Target]?

Knowledge (experience, observation, or information that you might draw on in your land management)

Where does the knowledge to manage [Target] come from?

Do you think the forest/park/field office) has the knowledge they need to effectively manage [Target]?

What kinds of additional knowledge do you need? How would you obtain that knowledge?

What kinds of monitoring does the forest/park/tribe/field office do on [Target]?

Do you think you have a good baseline understanding of the condition of [Target] on the land you manage?

How would your describe [target] and how it works in the Gunnison to someone who has never been here?

Climate Change and Targets

How do you think [Target] are currently or will be impacted by climate change?

How did climate change factor into the goals established [Target] in the current management plan?

Do you think your agency's current management goals are realistic in the context of climate change?

Questions that Come after the Target-Specific Questions

In the context of land management, what does the term 'climate adaptation' mean to you?

What kind of timescales does your agency consider when making decisions about the management of these two systems? (if they cite just short time frame follow up with: Does medium-term thinking, such as 10-20 year timeframes, come into play and if so how? How about even longer-term thinking, say 40-50 years out?)

How does thinking on these different timescales change the way the agency makes decisions? (or if they only cited one timeframe: How would thinking on these different timescales change the way the agency makes decisions?)

Uncertainty and Dynamic Ecosystems

There’s a lot of discussion of how to make management decisions in the context of uncertainty and incomplete knowledge of how systems such Greater Sagebrush and Spruce-Fir ecosystems are changing. I have a series of questions focused on trying to understand how current decision-making processes deal with uncertainty and change in ecosystem. I realize that these are difficult and complex questions, I am really interested in getting your perspective based on your experience within the agency.

What usually happens in existing planning and decision-making processes in situations with incomplete knowledge?

What if climate scientists and ecologists simply can’t provide very accurate or specific predictions about how particular systems will be impacted by climate change? In that context, what would enable you to make decisions?

Some people have suggested that in the context of uncertainty, we should manage for a range of future conditions. What do you think about that and what would enable that to happen in your agency?

Do you think that agency management and planning processes need to become more flexible in the face of the challenges we’ve discussed? How could that happen?

We are trying to develop a process to help agency staff and decision-makers integrate adaptation into management and decision-making. Do you have any thoughts on what would make that process most useful to you to help you to think about adaptation when you are making decisions in these systems?
