# Supplementary material for: Navigating Climate Adaptation on Public Lands: How Views on Ecosystem Change and Scale Interact with Management Approaches
Source: Environ Manage. 2020 Jul 29;66(4):614–28. doi: 10.1007/s00267-020-01336-y (PMC7522104; doi:10.1007/s00267-020-01336-y)
Supplement: Supplementary file 4 — Appendix 4 [file 267_2020_1336_MOESM4_ESM.pdf]

Appendix 4: Focus Group Guide

Questions for the four focus groups.

1. What’s your initial reaction to this scenario?
2. What’s particularly concerning about this scenario?
    a. How might this scenario impact management of public lands in the Gunnison?
    b. How might this scenario impact the local ranching community?
    c. How might this scenario impact the broader community?
3. What kind of problems/disputes/conflicts arise in this scenario?
4. What kinds of opportunities are present in this scenario?
5. What types of management strategies would you consider to deal with this scenario?
    a. How about management strategies to specifically address changes in sagebrush areas?
    b. How about management strategies to specifically address changes in spruce fir areas?
6. What are the barriers to implementing the strategies you’ve described?
7. Who needs to work together or collaborate to effectively respond?
8. How does this process help you think about making management decisions in the face of change and uncertainty?
