# Supplementary material for: Navigating Climate Adaptation on Public Lands: How Views on Ecosystem Change and Scale Interact with Management Approaches
Source: Environ Manage. 2020 Jul 29;66(4):614–28. doi: 10.1007/s00267-020-01336-y (PMC7522104; doi:10.1007/s00267-020-01336-y)
Supplement: Supplementary file 5 — Appendix 5 [file 267_2020_1336_MOESM5_ESM.pdf]

Appendix 5: Codes from interview and focus group analysis

Here we provide the codes used to analyze the transcripts from interviews and focus groups. These codes include both *a priori* and emergent codes and the lead author coded the materials in multiple iterations to ensure that all transcripts were coded for emergent codes.

Code:	Description
Adaptation	General discussion of social or ecological adaptation in response to climate change
Approach to Change	Responses to climate change and how people framed them
Barrier	Barriers to adaptation and management in the context of climate change
Climate Science	Views about and responses to climate science
Ecology	Ecological systems and interactions between different species within the terrestrial system
Extremes	Extreme events and natural hazards
Feedback	Feedback offered about the process at the end of the focus group sessions
Flexibility	Discussions of flexibility and inflexibility in terms of regulations, management, and individual actions
Grazing	Grazing practices, management, and impacts
Landscape change	Landscape transformation, state changes, and regime shifts
Management	Resource management practices, strategies, and objectives
No regrets	Framing and discussions of "no regrets" strategies
Recreation	Outdoor recreation activities, business operations, and relationship to resource management and public lands
Relationship with agencies	The relationships between different resource agencies, and the relationships between agencies and resource users
Sagebrush	Discussion of the sagebrush system, which was one of the targets for the broader research project
Sage grouse	Discussion of the Gunnison Sage grouse which were pending endangered or threatened listing
Scale and Boundaries	How temporal and geographic scale and boundaries impacted management, use of information, climate adaptation, and other elements of managing for change
Scientific Knowledge	How people talked about science, including how they used it, their thoughts about it, and barriers to using it
Spruce Fir	Responses directly about the spruce-fir system, which was one of the targets for the broader research project
Strategy	Strategies for how to adapt to climate change, particularly management approaches
Tensions	Conversations about local disagreements that highlighted differences of opinions. This focused on a number of local issues

	including endangered species issues, climate change politics, different approaches to management, etc.
Uncertainty	Responses that directly discussed future uncertainty or a range of future conditions
Vulnerability	Discussions of both ecological vulnerabilities and social vulnerabilities due to climate change
Water Resources	Specific responses about water resources and use in the Basin
Wildlife	Discussions of wildlife in the Basin, primarily how they would be impacted by climate change and how to manage populations in that context
